# House dust mite‐specific immunotherapy with two licensed vaccines: Outcome under clinical routine conditions

**DOI:** 10.1002/iid3.141

**Published:** 2017-03-05

**Authors:** Vera Mahler, Christian Klein, Angelika Sager, Jürgen Zimmermann

**Affiliations:** ^1^ Hautklinik Universitätsklinikum ErlangenErlangenGermany; ^2^ Novartis Pharma GmbHNürnbergGermany; ^3^ LETI Pharma GmbHWittenGermany

**Keywords:** Depigoid^®^, house dust mite, specific immunotherapy

## Abstract

**Introduction:**

House dust mite (HDM) allergens are major causes for the development of allergic diseases. A disease modifying effect and clinical benefit of allergen immunotherapy (AIT) has been demonstrated in a number of clinical trials. Clinical trials, however, are carried out in selected populations under specific conditions based on inclusion and exclusion criteria and may not represent the entire patient population from medical practice. Objective of this study conducted in patients with HDM allergy was to systematically collect information about the benefit of AIT under clinical routine conditions.

**Methods:**

In this prospective, multi‐center non‐interventional study, 220 patients (117 adults, 103 children) with HDM allergy receiving subcutaneous AIT with Depigoid^®^ were monitored for 2 years. Organ‐specific key symptoms, health‐related quality of life (QoL), and the use of concomitant anti‐allergic medication were assessed at baseline and after 12 and 24 months. Effectiveness and tolerability of the AIT was assessed by physicians and patients. Occurrence of adverse events (AEs) was continuously monitored.

**Results:**

Two hundred and nineteen patients (116 adults, 103 children) were evaluated. A major improvement of the total symptom‐score was observed after 24 (12) months in 76% (72%) and 80% (79%) of adults and children, respectively, accompanied by a reduction in concomitant anti‐allergic medication and a pronounced improvement in QoL. The effectiveness and tolerability of the AIT was estimated as very good/good by 80–90% of physicians and patients. AEs were observed in 4/117 adults (3.4%) and in 7/103 children (6.8%). Serious AEs were reported in three adults and one child: A grade‐II anaphylactic reaction (one adult) controlled by oral antihistamines (no hospitalization) classified as “definitely,” three others as not (2) or possibly (1) drug‐related.

**Conclusions:**

The data collected from 220 patients confirm the efficacy, tolerability/safety, and acceptance of AIT with Depigoid^®^ in adults and children with HDM allergy under routine clinical conditions.

## Introduction

House dust mite (HDM) allergens are major causes for the development of allergic diseases such as allergic rhinitis and asthma. Approximately 50% of patients with a clinical diagnosis of allergic rhinitis have a sensitization to HDM allergens 1. HDM are prevalent in homes in Europe with large variations across countries, ranging from 0.5% in Iceland to nearly 100% in parts of Spain 2. Taxonomically, approximately 50,000 species have been described 3, however, the most common mite allergens involved in type I‐allergies are derived from *Dermatophagoides pteronyssinus* and *Dermatophagoides farinae*. Both species are widely common in Europe 2. Treatment strategies for controlling HDM allergy consist of allergen avoidance, symptomatic treatment, and allergen‐specific immunotherapy. Measures to avoid HDM such as the use of acaricides and extensive bedroom‐based environmental control programs may be of some benefit in reducing rhinitis symptoms, although the evidence is not considered strong 4. The administration of symptomatic medication, that is, antihistamines and corticosteroids, can control symptoms but does not affect either the underlying cause or the progress of the disease. Allergen immunotherapy (AIT), on the other hand, is able to modify the natural course of the disease and thereby reduces symptom severity and requirement of symptomatic medication 5, 6. The efficacy of AIT seems to be dose‐dependent with higher doses achieving a higher clinical efficacy 7. However, the injection of high doses of potent standardized allergen vaccines is also risk factor for systemic reactions including anaphylaxis 8. Therefore, glutaraldehyde‐modified allergen extracts were developed, aiming at better safety profiles than unmodified vaccines while retaining clinical efficacy 9, 10, 11. More recently, these modified allergen extracts were further improved by a depigmentation step before polymerization 12, 13. These polymerized depigmented extracts are significantly less allergenic than the corresponding native extracts and allow administration of larger doses 12. In 2005, the allergen extracts Depigoid^®^ *D. pteronyssinus* and Depigoid^®^ House‐Dust‐Mite‐Mixture (50% of *D. pteronyssinus* and 50% of *D. farina*e) have been approved in Germany for the treatment of IgE‐mediated allergies caused by HDM. A number of clinical studies (one open, parallel, controlled, and random‐allocated, two double‐blind placebo‐controlled) have shown that treatment with the allergen extracts is safe and efficacious 14, 15, 16. Clinical trials are, however, carried out under special conditions based on inclusion and exclusion criteria and the obtained data may not represent the whole patient population from medical practice. The objective of this non‐interventional study was, therefore, to obtain data on safety, tolerability, and efficacy of treatment with Depigoid^®^ *D. pteronyssinus* and Depigoid^®^ *HDM mixture* in patients treated according to medical practice under clinical routine conditions to evaluate the benefits and potential risks in routine use.

## Methods

### Study design and treatment

In this prospective, multi‐center, non‐interventional study patients with allergy (asthma, rhinitis, or both) to HDM treated according to the standards of medical practice with Depigoid^®^ *D. pteronyssinus* or Depigoid^®^ *HDM mixture,* between February 2011 and December 2013, were documented by physicians in 70 study centers distributed across Germany. For both Depigoid^®^ *D. pteronyssinus* and Depigoid^®^ *HDM mixture*, two recommended treatment regimen according to the Summary of Product Characteristics (SmPC) exist: (1) a 4‐week build‐up regimen with escalating doses (2, 5, 20, and 50 DPP) administered at weekly intervals followed by a maintenance regimen with administration of 50 DPP every 4 weeks. (2) Alternatively, a quick build‐up dosing regimen 17, 18 (day 1: 20 and 30 DPP with a 30‐min interval; further dosages of 50 DPP to be applied in 4 week intervals).

These two alternative treatment regimen could be modified as deemed appropriate by the physician.

### Patients

Patients ≥5 years of age with a symptomatic allergy (asthma, rhinitis, and/or conjunctivitis) in line with confirmed IgE‐mediated immediate hypersensitivity (type I), a positive result from an appropriate skin allergy test and, if available, in vitro specific serum IgE against house dust mites who had no contraindications to treatment with Depigoid^®^ *D. pteronyssinus* or Depigoid^®^ *HDM mixture* as listed in the SmPC were eligible for this study. Written consent regarding the documentation of data in the context of this study and regarding the inspection of the patient's medical records for source data verification was a prerequisite for inclusion of the patient in the study.

### Data collection and documentation of adverse events

**Figure 1:**
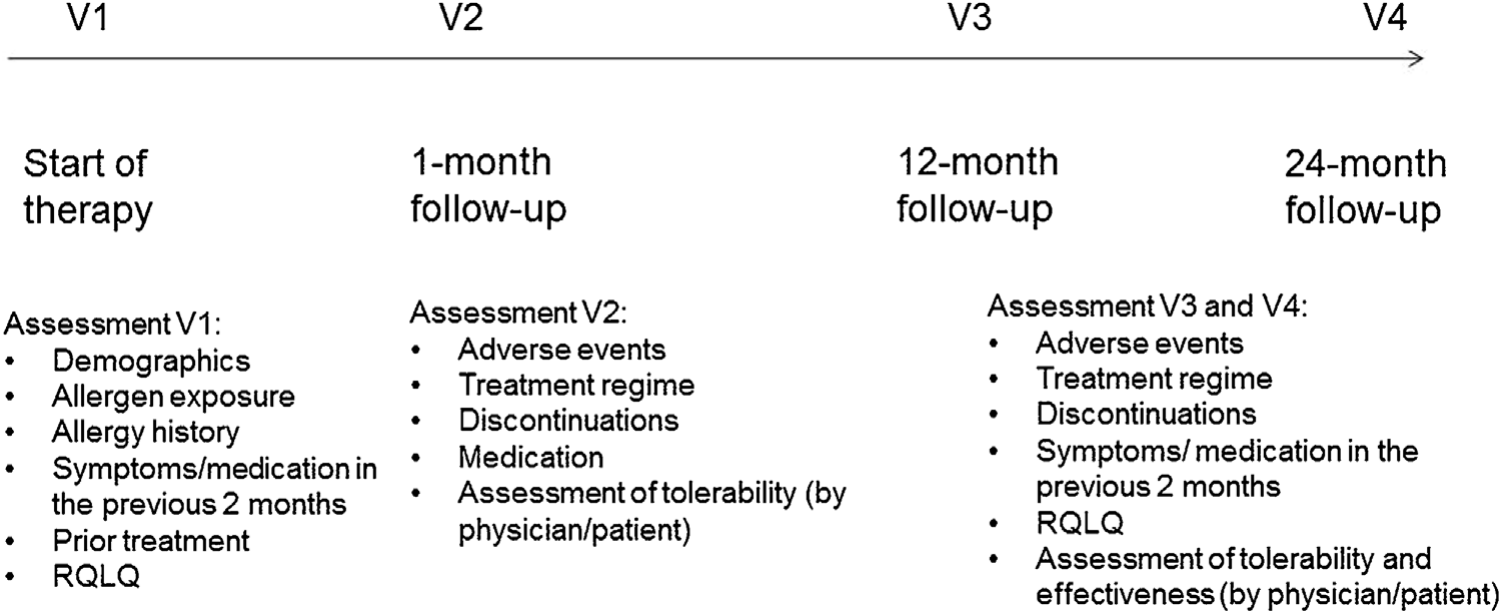
Study diagram. RQLQ, Rhinoconjunctivitis Quality of Life Questionnaire (RQLQ); V, visit.

The time schedule and variables of the study are illustrated in Figure 1.

At visit 1 (V1), demographic data and information on the exposure to allergens as well as data on the allergic history were recorded. Symptoms and medication used in the previous 2 months were recorded retrospectively.

Organ‐specific sum scores for the three main symptoms (ocular, nasal, and bronchial) and the four specific asthma symptoms (dyspnea, tightness in the chest, wheezing, coughing) were each assessed on a 4‐point scale from 0 to 3 (no/mild/moderate/severe) for each of the seven single symptoms. Thus, total sum scores are potentially covering values from 0 up to 21. The clinical assessment of the severity of symptoms is related to categories of “0 = no disorders at all,” “1 = mild” (disorders present, but not bothersome), “2 = moderate” (disorders bothersome, but not restrictive or intolerable), and “3 = severe” (disorders restrictive and/or intolerable).

The different types of anti‐allergic medication that had been used (local antihistamines, systemic antihistamines, inhaled corticosteroids, oral corticosteroids, nasal corticosteroid sprays, corticosteroids eye drops, inhaled beta‐2‐agonists) were each documented on a scale from 0 to 3 (not at all/rarely/occasionally/frequently used) and were individually as well as combined as sum score of concomitant anti‐allergic medications evaluated before and during the course of AIT.

Health‐related quality of life data were obtained by means of the Rhinoconjunctivitis Quality of Life Questionnaire (RQLQ) according to Juniper, E. 19. The RQLQ is a validated questionnaire which has 28 questions in seven domains (sleep, non‐rhinoconjunctivitis symptoms, practical problems, nasal symptoms, eye symptoms, activity limitations, and emotional function). Patients are asked to recall impairments experienced during the previous week and to respond to each item on a 7‐point scale (0 = no impairment, 6 = maximum impairment). The overall RQLQ score is the mean of all 28 responses and higher scores reflect lower quality of life. A difference of 0.5 points in the RQLQ score is generally regarded as clinically significant 19. Age appropriate paper versions were used: pediatric RQLQ (≥5 but <12 years of age); adolescent RQLQ (≥12 but <18 years of age); adult RQLQ (≥18 years of age)). The questionnaires were completed by the patients themselves or by their parents, if appropriate.

At the end of the 4‐week built‐up period (weekly injections) and 1 month after the start of therapy, patients came back for study‐related assessments in visit 2 (V2). At V2, physicians documented treatment regimen, date and reasons for premature discontinuation of therapy and use of anti‐allergic medication. Treatment tolerability was assessed by the physician and the patient on a scale from 1 to 4 (1 = very good, 2 = good, 3 = moderate, 4 = bad).

At visit 3 and 4 (V3/V4), physicians documented the treatment regime as well as date and reasons for premature discontinuation. Furthermore, symptoms and medication in the previous 2 months were recorded and compared with the data obtained at V1. Patients were once again asked to fill in the RQLQ and to assess tolerability as well as effectiveness of the therapy (scale of 0–3: very good/good/moderate/bad). Tolerability and effectiveness of the treatment were also assessed by the physician.

In the course of the study, any adverse event (AE) had to be documented by the physician. An AE was defined as any untoward medical occurrence in a subject to whom a medicinal product was administered, irrespective of whether the event was suspected to be causally related to a medicinal product. AEs were specified by the physician in the case report form (CRF) as diagnosis and assessed by severity (mild/moderate/severe), causality (verified/likely/possible/unlikely/no casual relation/not assessable), outcome (recovered/not recovered/fatal/life‐threatening/stationary treatment/inability to work or invalidity/congenital anomaly or birth defect/unknown), and whether it was serious (yes/no). Serious adverse events (SAE) were all events that were fatal, life‐threatening, resulted in inpatient hospitalization or a prolongation of existing hospitalization, resulted in inability to work, persistent or significant disability, or invalidity, resulted in a congenital anomaly or a birth defect or were otherwise judged medically relevant, that is, affected the patient considerably, but did not meet any of the above criteria.

### Statistical analysis

Statistical analyses were carried out using SAS software, version 9.2 (SAS Institute, Cary, North Carolina). All data were analyzed descriptively. The results, including exploratory *p*‐values (*p*‐values < 0.05 are regarded as nominally significant), were interpreted descriptively. No adjustment for multiple testing was performed. All analyses were performed by treatment (Depigoid^®^ *D. pteronyssinus* or Depigoid^®^ *HDM mixture*) and by age class (adult or child). Patients were classified by age according to their age at study entry (<18 years vs. ≥18 years). An exception was made only for the analysis of RQLQ data, where the patients were classified according to the RQLQ version used. Data of patients were analyzed for two populations: the safety population which included all patients enrolled who received at least one dose of the treatment and the full analysis set (FAS) which included all patients of the safety population for whom at least one follow‐up documentation was available.

Shift tables (baseline vs. 12 or 24 months) were prepared to describe changes in the severity of main symptoms and specific asthma symptoms and the frequency of use of concomitant anti‐allergic medication. McNemar tests were used to compare the frequency of improvements and worsening of symptoms.

Missing values were generally not replaced. However, the key analyses of symptoms and concomitant anti‐allergic medications at month 24 were performed using the last observation carried forward (LOCF) approach for replacement of missing data due to a relatively high number of missing values at month 24.

This study and the related observational plan has been notified to health authorities and approved by the responsible ethics committee (ethics committee of the medical faculty of the Friedrich‐Alexander University Erlangen‐Nuremberg).

## Results

### Patients

Two hundred and twenty patients (117 adults, 103 children) received specific immunotherapy with Depigoid^®^ *D. pteronyssinus* or Depigoid^®^ *HDM mixture*. Two hundred and nineteen of the 220 patients in the safety population had at least one follow‐up examination documented and qualified, therefore, for the FAS. Eighty seven percent (*n* = 196) of the study patients were documented with data for 12 months of AIT and 75% (*n* = 169) of the patients have completed the full 24 months treatment course. Almost all patients, except for seven (two adults, five children) were treated with Depigoid^®^ *HDM mixture*. Therefore, the following description of results does not differentiate between the two treatments.

The patients’ characteristics at the start of the study are summarized in Table 1. In summary, this patient collective was very heterogeneous and included patients of different age, weight, and height and represented well the “real‐life” population in medical practice. The living situation was equally balanced, with approximately one‐third of the patients each living in the city center, the suburban area, or the rural area. At baseline, most patients suffered from nasal symptoms (99% of adults; 92% of children), while ocular and bronchial symptoms were less common (adults: 85% and 63%, respectively; children: 60% and 64%, respectively). The mean total sum score of all seven symptoms (the three main symptoms and four asthma‐specific symptoms) was 7.5 ± 4.0 in adults and 7.2 ± 4.4 in children indicating that patients, in general, suffered from mild to moderate allergic symptoms even though some patients suffered from severe symptoms as indicated by the wide range of scores.

**Table 1:** Patient characteristics at baseline

Parameter	Adults (*n* = 116)	Children (*n* = 103)
Sex (n [%])		
Male	55 (47.4)	60 (58.3)
Female	60 (51.7)	43 (41.7)
Missing	1 (0.9)	–
Mean age (years) ± SD	37.8 ± 15.1	11.2 ± 3.1
Range (years)	18–82	5.4–18
Mean height (cm) ± SD	172.1 ± 9.0	146.2 ± 17.4
Range (cm)	155‐194	110‐190
Mean weight (kg) ± SD	75.4 ± 14.6	43.6 ± 17.2
Range (kg)	50–136	19–98
Mean BMI (kg/m^2^) ± SD	25.4 ± 4.4	19.5 ± 4.0
Range (kg/m^2^)	16.8–43.9	12.8–30.2
Living situation (n [%])		
City center	41 (35.3)	38 (36.9)
Suburban	32 (27.6)	27 (26.2)
Rural	30 (25.9)	32 (31.1)
Missing	13 (11.2)	6 (5.8)
Remedial already measures taken (n [%])		
Anti‐allergy mattress	23 (19.8)	24 (23.3)
Anti‐allergy bedclothes	32 (27.6)	47 (45.6)
Mattress encasing	38 (32.8)	70 (68.0)
Mattress vacuum cleaner	4 (3.4)	1 (1.0)
Special filter for vacuum cleaner	5 (4.3)	17 (16.5)
Air purifier	–	–
Physician's assessment of exposure to domestic mites (n [%])		
Low	11 (9.5)	20 (19.4)
Moderate	66 (56.9)	49 (47.6)
High	15 (12.9)	16 (15.5)
Unclear	20 (17.2)	14 (13.6)
Missing	4 (3.4)	4 (3.9)
Interval between diagnosis and start of therapy (years)		
Mean ± SD	2.2 ± 4.0	2.8 ± 2.7
Median (range)	0.6 (0.0–22.7)	2.2 (0.0–12.7)
Total sum score of symptoms^a^		
Mean ± SD	7.5 ± 4.0	7.2 ± 4.4
Median (range)	7 (0–19)	6 (0–20)
Total sum score of allergic^b^ medications		
Mean ± SD	5.1 ± 4.4	5.5 ± 4.4
Median (range)	4 (0–18)	5 (0–19)

BMI, body mass index; SD, standard deviation.

^a^ Total sum scores of symptoms could range from 0 = no symptoms to 21 = all three main symptoms and four specific asthma symptoms severe.

^b^ Total sum scores of anti‐allergic medications could range from 0 = no medication to 21 = all seven types of medication used frequently.

### Effectiveness

**Figure 2:**
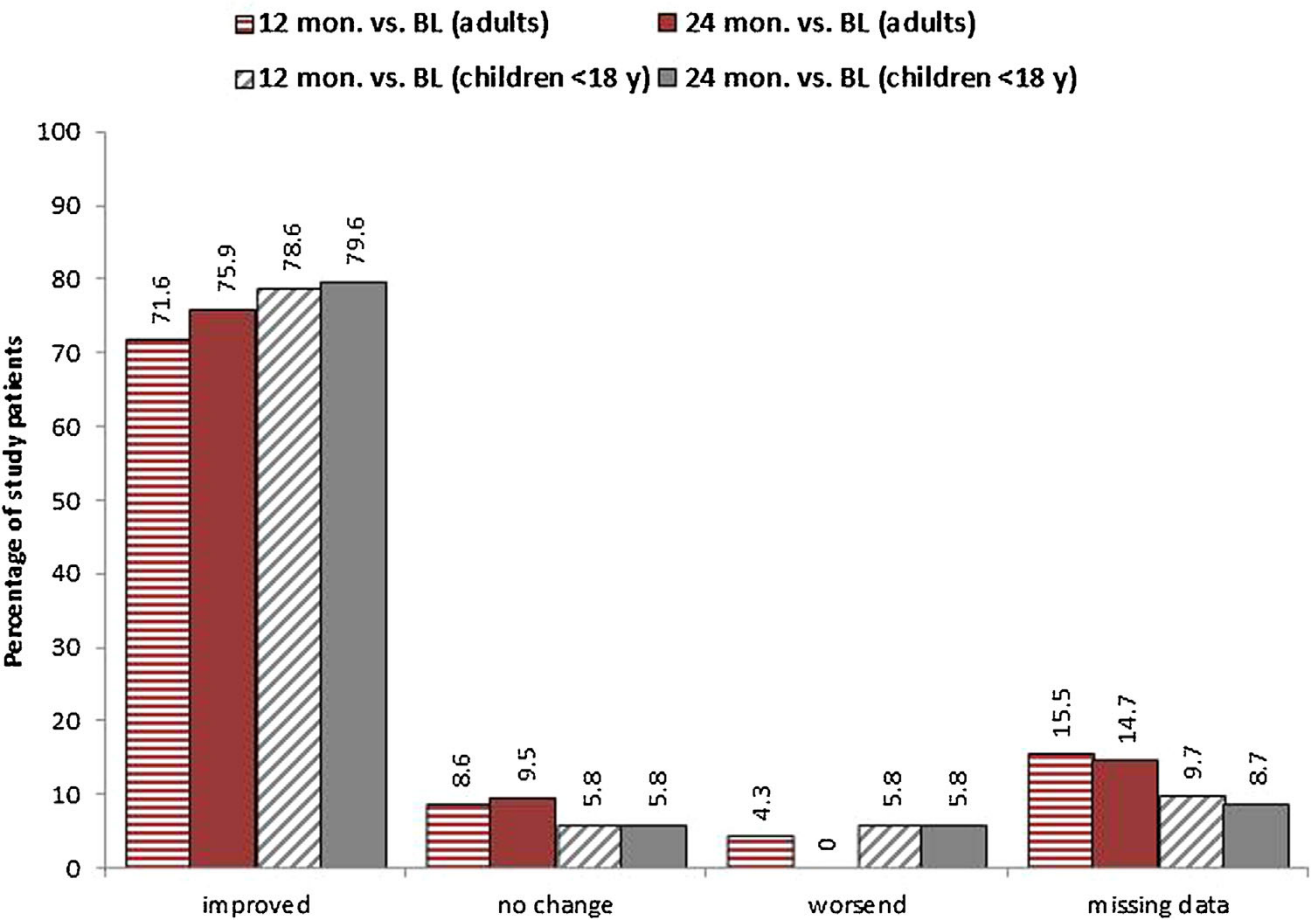
Total sum score of symptoms: Percentage of study patients with changes during 12 and 24 months of treatment with Depigoid^®^ (*N* = 219 [116 adults, 103 children]).

**Figure 3:**
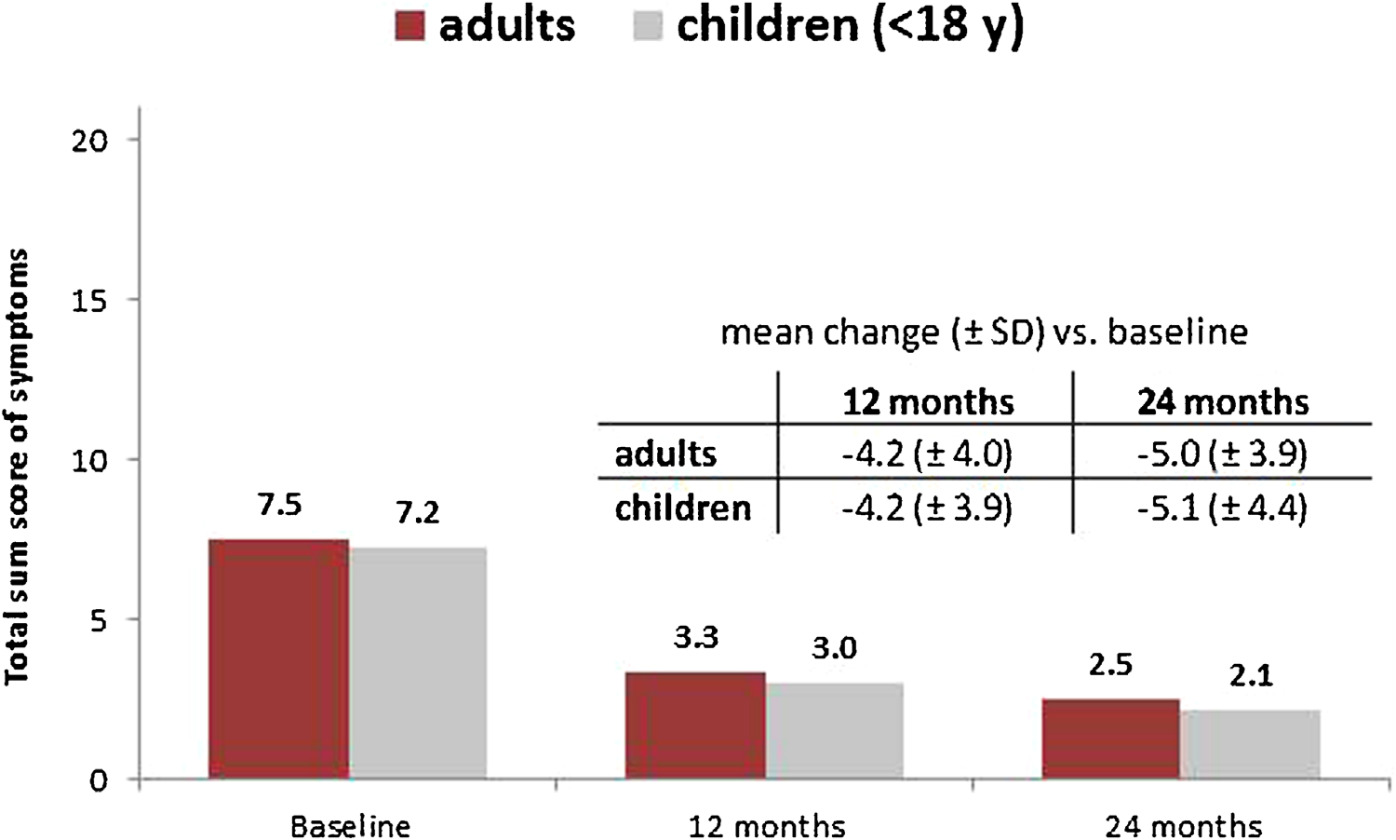
Total sum score of symptoms (mean): Changes of scores values in adults (*N* = 116) and children (*N* = 103) during 12 and 24 months of treatment with Depigoid^®^. Mean change values (±SD) versus baseline after 12 and 24 months. Evaluation of three organ‐specific symptoms (related to nose, eyes, and lung) and four asthma‐specific symptoms according to severity (0–3); max. 21 score points [severity ranges: (0) = no disorders, (1) mild = disorders present/not bothersome, (2) moderate = bothersome/not disabling or intolerable, (3) severe = disabling and/or intolerable].

Overall, an improvement of the total sum score of symptoms after 24 (12) months was observed in 76% (72%) and 80% (79%) of adults and children, respectively (Fig. 2). On average, the total sum score decreased by 4–5 points over the observation period from approximately seven points at baseline to approximately three (12 months) and two points (24 months) (*p* < 0.001 for both adults and children [at 12 and at 24 months, also]) (Fig. 3). Individually, all main symptoms (nasal, ocular, bronchial) improved over the course of the study (*p* < 0.001 for both adults and children at each time point and for each symptom).

The use of concomitant anti‐allergic medication by both adults and children decreased from a score of 5–6 at baseline to a score of 2–3 at 12 and 24 months (*p* < 0.001 for both adults and children at 12 and 24 months).

In the majority of cases, the physician assessed the effectiveness of treatment with Depigoid^®^ as “good” or “very good” (adults: 78.5% [12 months]; 61.2% [24 months]; children: 89.3% [12 months]; 70.9% [24 months]). Equally positive assessments were obtained from the patients themselves (adults: 79.4% [12 months]; 60.3% [24 months]; children: 87.3% [12 months]; 69.9% [24 months]). The numerical differences seen between months 12 and 24 are due to a high number of missing values at month 24 (34–35% for adults and 25% for children) and assumedly not due to a loss of effectiveness since the stratification of the data by study completion status showed that the baseline sum scores of concomitant anti‐allergic medications were, on average, roughly similar in patients who completed 24 months and in patients who discontinued prematurely. The same applied to the change 12 months versus baseline.

### Health‐related quality of life

**Figure 4:**
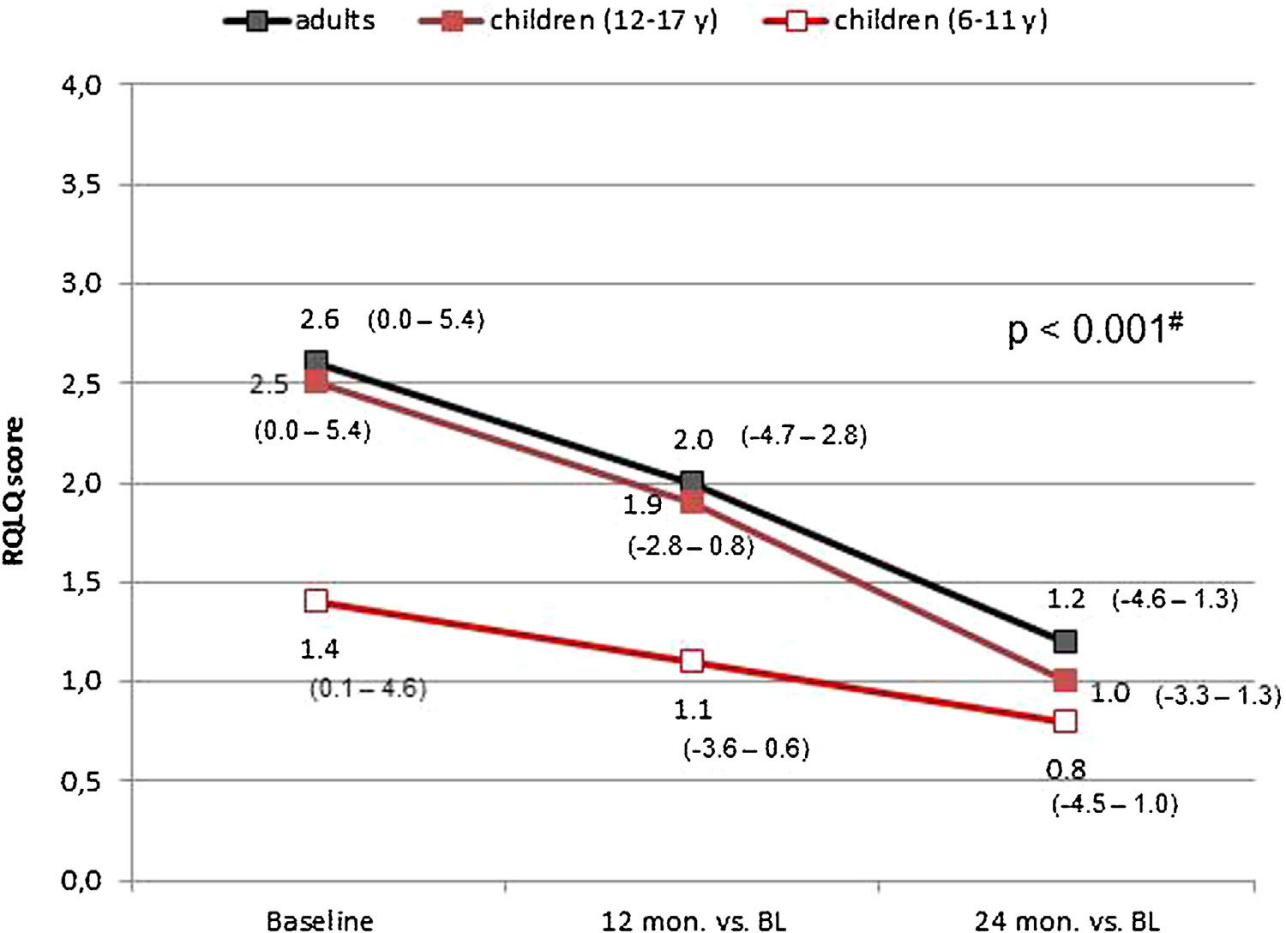
Impact of Depigoid^®^ treatment on health‐related quality of life evaluated by Rhinoconjunctivitis Quality of Life Questionnaire (RQLQ). Absolute changes of RQLQ median values (ranges in brackets) from baseline to month 24 in adults (*N* = 109) and children aged 12–17 (*N* = 35) and 6–12 (*N* = 57) years, respectively. Possible score values ranging from 0 (no limitation) to 6 (severe limitation). A difference of 0.5 in the RQLQ score is considered as clinically significant. ^#^ *p*‐Values of <0.001 indicating statistical significant changes from baseline for all groups at 12 and 24 months.

A comparison of changes in quality of life from baseline to the end of study at 24 months in adults and adolescents showed a pronounced improvement during the course of the study for all seven domains of the RQLQ as well as the total score: in the course of the treatment period the total RQLQ score dropped from 2.6 at baseline (BL) to 1.2 in adults and from 2.5 (1.4) to 1.0 (0.8) in children at the age of 12–17 and 6–12 years, respectively (Fig. 4). A difference of 0.5 in the RQLQ score is generally regarded as clinically relevant and was statistically significant for both adults and children at 12 and 24 months (*p* < 0.001) 19. The average improvement of RQLQ total and domain scores was less pronounced in younger children (5–12 years of age). It has to be taken into consideration, however, that the mean baseline values were also lower in this subgroup.

### Safety and tolerability

During the 24‐month study period, AEs were observed in four of the 117 adults (3.4%) and in seven of the 103 children (6.8%). Data are shown in Table 2. All non‐serious AEs were related to the study drug and were injection site conditions such as erythema, swelling, and pain.

**Table 2:** Summary of adverse events

Parameter	Adults (*n* = 117), *N*_patients_ (%)	Children (*n* = 103), *N*_patients_ (%)
AE, total	4 (3.4)	7 (6.8)
Non‐serious AE	1 (0.9)	5 (4.9)
Drug‐related	1 (0.9)	5 (4.9)
Not related	–	–
SAE	3 (2.6)	1 (1.0)
Drug‐related^a^	2 (1.7)	–
Not related	1 (0.9)	1 (1.0)
No information on seriousness^b^	–	1 (1.0)

AE, adverse event; SAE, serious adverse event.

^a^ SAE including one case with two episodes of exacerbations of infection in a COPD patient—one before (not related) and one after administration of Depigoid^®^ (possibly related).

^b^ AE without information on seriousness: Mild pruritus lasting for 1 day, resolved, no action taken, drug‐related.

Three adults and one child experienced SAEs. In a 33‐year‐old male, an anaphylactic reaction occurring after administration of the first dose (2 DPP) of Depigoid^®^ *HDM mixture* was reported. The reaction was successfully treated with oral antihistamines without need for hospitalization; due to the benign progress, this reaction was considered as grade II following EAACI criteria. The investigator classified this SAE as “definitely” drug‐related and the patient was excluded from further treatment with Depigoid^®^ and has been withdrawn from the study. A 48 year‐old male patient with a medical history of COPD experienced two 4‐day episodes of exacerbation of an infection. The first episode began 3 days before administration of a (maintenance) dose of Depigoid^®^ *HDM mixture* and was classified as not related to the study treatment. The second episode started 6 days later, that is, 3 days after administration of Depigoid^®^, and was classified as “possibly related.” The patient was admitted to hospital. Both events were resolved without sequelae.

Non‐drug‐related SAEs were experienced by a 55‐year‐old female who had pneumonia and an 11‐year‐old boy who developed sarcoidosis.

Tolerability after 12 and 24 months was assessed by physicians as “good” or “very good” in 81.9% (64.7%) of adults and 91.3% (73.8%) of children; the corresponding patient's assessments was “good”/“very good” in 83.6% (66.4%) of adults and 91.3% (74.4%) of children. The numerical decrease seen between months 12 and 24 is due to a large number of missing values at month 24 (32–35% for adults and 25% for children) and not equivalent to a decrease in tolerability as no increase of “moderate” or “poor” tolerability was observed.

## Discussion

Despite numerous clinical trials and meta‐analyses proving allergen immunotherapy efficacious, it remains underused and is estimated to be used in less than 10% of patients with allergic rhinitis or asthma worldwide 20. Relevant allergens are major contributors to the safety and efficacy of the allergenic extracts used for AIT. Most of the available data address mites, selected pollens, and animal dander, whereas less is known about the efficacy and safety of mold or cockroach allergens 20.

In this prospective, multi‐center non‐interventional study, data were recorded on 220 patients (of whom 219 were treated and had at least one follow‐up examination) who were allergic to house dust mites and treated in medical practice under real life conditions with either Depigoid^®^ *D. pteronyssinus* or Depigoid^®^ HDM mixture.

In concordance with the non‐interventional set‐up of this study, the number of missing values increased from month 12 to 24: Approximately 90% of the patients were documented 12 months after the start of therapy and approximately 75% after 24 months. None of the patients were reported to have discontinued therapy due to an adverse drug reaction. Additional data analyses stratified by study completion status (not shown in the paper) revealed, that the number of patients lost to follow‐up after the first year was independent of the treatment outcome: Patients documented for only 12 months showed the same 12 months efficacy and tolerability (vs. baseline) as patients completing the full 24 month study course. However, from this finding it cannot be concluded that AIT can be generally terminated after one year of treatment since additional and long‐lasting benefits of a longer treatment period may be expected 21.

A usual LOCF (i.e., Last Observation Carried Forward) analysis was conducted to include all available outcome data irrespective of the study completion status.

Overall, treatment adherence was comparatively good as other studies have shown that treatment persistence and compliance are frequently reducing over time 22, 23. A German study analyzing health insurance data, for example, showed that only 45%/24% of patients receiving subcutaneous AIT continued with their therapy in the second year and third year, respectively 22. A shorter therapy regimen 22, better patient education, strict follow‐ups as well as a regular contact between patient and physician could increase adherence 22.

Less than 4% of the adults and 7% of the children reported AEs indicating a good safety profile. Two patients experienced AEs that were classified as serious adverse drug reactions (one experienced a drug‐related anaphylactic grade II reaction and the other a possibly drug‐related COPD‐exacerbations of an infection). All other (possibly) treatment‐related AEs were transient, mild to moderate injection site conditions such as erythema, pain, and swelling. This good safety profile observed in the study is in concordance with other studies where patients were treated with Depigoid^®^ *D. pteronyssinus* or Depigoid^®^ House‐Dust‐Mite‐Mixture 14, 15, 16, 24. In a prospective, multi‐center non‐interventional study on 768 patients with allergic rhinoconjunctivitis and allergic asthma specific immunotherapy with different Depigoid^®^ allergen extracts (Depigoid^®^ *HDM mixture*, Depigoid^®^ *D. pteronyssinus*, and Depigoid^®^ containing allergens of either grasses, trees, weeds, or others) was carried out. The best safety results were observed for the 132 patients vaccinated against HDM with only two local and four grade 1 systemic reactions 24. Taken together with the novel results of this study, the data show that treatment with Depigoid^®^ *D. pteronyssinus* or Depigoid^®^ *HDM mixture* has a good safety profile under real‐life clinical conditions.

In general, clinical efficacy under daily clinical conditions seemed to be good and in concordance with previous studies 15, 24. Over the course of the study an improvement of symptoms was observed in the majority of patients while worsening of symptoms was rare. This was accompanied by a reduction in concomitant anti‐allergic medication and an improvement in quality of life. These effects were seen in both children and adults indicating that patients of all ages profit from the treatment. Overall, the patient population consisted of a diverse group of subjects with a broad demographic pattern. Participants differed not only in age but also height, weight, disease duration, and severity which reflects real life of medical practice. This is in contrast to clinical studies where the focus lies on selected patient groups which may not represent the general population or patients seen in normal clinical practice.

In summary, the data collected in this study showed that treatment with Depigoid^®^ *D. pteronyssinus* or Depigoid^®^ House‐Dust‐Mite‐Mixture was effective, well tolerated, and safe in adults as well as in children treated in medical practice under clinical routine conditions.

## Author Contributions

Vera Mahler substantially contributed to the conception and design of the study, analysis and interpretation of data, drafting and revising the article critically for important intellectual content and gave final approval of the version to be published. Christian Klein substantially contributed to the conception and design of the study, analysis and interpretation of data, revising the article critically for important intellectual content and gave final approval of the version to be published. Angelika Sager substantially contributed to the conception and design of the study, revising the article critically for important intellectual content and gave final approval of the version to be published. Jürgen Zimmermann substantially contributed to the acquisition of data and analysis and interpretation of data, drafting and revising the article critically for important intellectual content, and gave final approval of the version to be published.

## Conflict of Interest

Vera Mahler has received honoraria as a speaker on allergy treatment from Novartis, ALK, HAL, Allergopharma, and Bencard, additionally as a scientific advisory board member from Novartis and ALK. She reports personal fees (as scientific advisor) from Novartis Pharma GmbH during the conduct of the study; personal fees (as a speaker) from Novartis outside the submitted work. Christian Klein and Jürgen Zimmermann are employees of Novartis Pharma GmbH, which was sponsor during the conduct of the study. Angelika Sager is employee of LETI Pharma GmbH, which is producer of the allergen vaccines used during the conduct of the study.
